# Supplementary figures and images for: Anti-Inflammatory and Immune Modulatory Effects of Synbio-Glucan in an Atopic Dermatitis Mouse Model
Source: Nutrients. 2021 Mar 26;13(4):1090. doi: 10.3390/nu13041090 (PMC8067118; doi:10.3390/nu13041090)

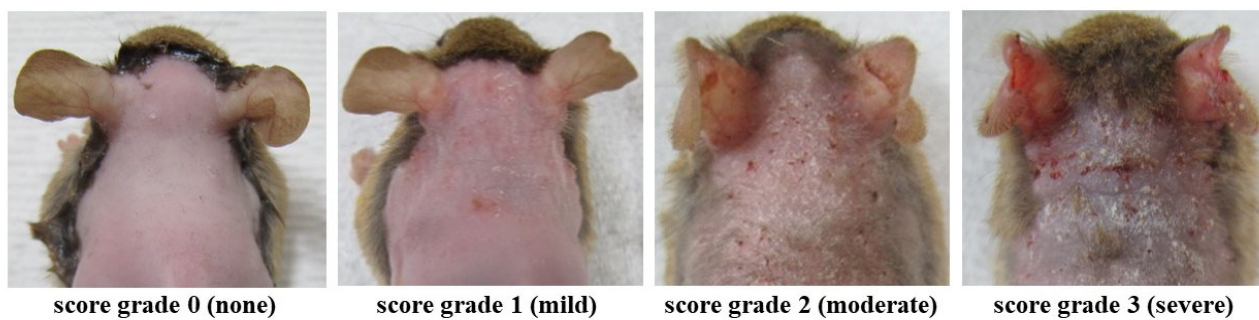

Figure S1. Scoring of skin lesions.

Supplement: Supplementary file 1 [file nutrients-13-01090-s001.pdf]
